# Olfactory system-inspired electronic nose system using numerous low-cost homogenous and hetrogenous sensors

**DOI:** 10.1371/journal.pone.0295703

**Published:** 2023-12-08

**Authors:** Sang Woo Lee, Byeong Hee Kim, Young Ho Seo

**Affiliations:** 1 Department of Smart Health Science and Technology, Kangwon National University, Chuncheon, Gangwon-do, Republic of Korea; 2 Department of Mechatronics Engineering, Kangwon National University, Chuncheon, Gangwon-do, Republic of Korea; Tokai University, JAPAN

## Abstract

This paper presents an electronic nose system inspired by the biological olfactory system. When comparing the human olfactory system to that of a dog, it’s worth noting that dogs have 30 times more olfactory receptors and three times as many types of olfactory receptors. This implies that the number of olfactory receptors could be a more important parameter for classifying chemical compounds than the number of receptor types. Instead of using expensive precision sensors, the proposed electronic nose system relies on numerous low-cost homogeneous and heterogeneous sensors with poor cross-interference characteristics due to their low gas selectivity. Even if the same type of sensor shows a slightly different output for the same chemical compound, this variation becomes a unique signal for the target gas being measured. The electronic nose system comprises 30 sensors, the e-nose had 6 differing sensors with 5 replicates of each type. The characteristics of the electronic nose system are evaluated using three different volatile alcoholic compounds, more than 99% of which are the same. Liquid samples are supplied to the sensor chamber for 60 seconds using an air bubbler, followed by a 60-second cleaning of the chamber. Sensor signals are acquired at a sampling rate of 100 Hz. In this experimental study, the effects of data preprocessing methods and the number of sensors of the same type are investigated. By increasing the number of sensors of the same type, classification accuracy exceeds 99%, regardless of the deep learning model. The proposed electronic nose system, based on low-cost sensors, demonstrates similar results to commercial expensive electronic nose systems.

## Introduction

The concept of mimicking the biological olfactory mechanisms was first proposed by Dodd [1]. Subsequently, in 1994, Gardner et al. proposed a new definition for the artificial olfactory system, i.e., the electronic nose (e-nose). The e-nose is an intelligent sensor array system designed to mimic biological olfactory functions. It comprises a gas delivery system for acquiring the odor patterns of complex volatile organic compounds (VOCs), a chemical sensor array, and a pattern analysis system for detecting and classifying odor patterns [2]. The e-nose has proven to be effective for distinguishing different odors and has been applied in various fields, including food analysis [3–11], medical diagnostics [12–16], environmental monitoring [17–23], and quality identification [24–26]. Gas sensors used in the e-nose system include metal oxide semiconductor (MOS) [3, 5, 16, 17, 24], conducting polymer (CP) [7, 12, 15], quartz crystal microbalance (QCM) [4, 6, 13], surface acoustic wave (SAW) [14], and optical sensors [25]. According to Alphus D. Wilson et al. [27], these various gas sensors had unique advantages and disadvantages due to their sensing mechanism. For examples, MOS sensors feature extremely high sensitivity, a limited detection range, and fast response and recovery times for low-molecular-weight compounds. However, MOS sensors require specific temperature maintenance for operation and consumes a significant amount of power. The advantages of CP sensors include room-temperature operation, sensitivity to a wide range of VOCs, short response time, a wide range of coatings, low cost, and resistance to sensor poisoning. However, CP sensors have several disadvantages, including sensitivity to humidity and temperature, vulnerability to sensor overload, and a limited sensor lifetime. QCM sensors offer the advantages of high precision, wide sensor range, and high sensitivity. However, QCM sensors present the disadvantages of complex circuitry, low signal-to-noise ratio, humidity, and temperature sensitivity. SAW sensors exhibit high sensitivity, fast response times, various sensor-range coatings, compact manufacturing, low cost, and sensitivity to almost all gases. However, SAW sensors feature complex circuitry, temperature sensitivity, and limited gas selectivity owing to the polymer film sensor coatings. Optical sensors are highly sensitive, multiparametric, and can identify individual compounds in a mixture. However, their disadvantages include complex sensor array systems, higher operating costs, and low portability due to the use of delicate optical and electrical components [27]. The main issues in improving the performance of sensors in existing e-nose systems are discreteness, drift, and disturbance. Discreteness affects sensor reproducibility by causing slight differences in sensor responsiveness due to the inherent variability in the sensor manufacturing process [28]. Drift is due to sensor aging, temperature, humidity, pressure, sensor poisoning, etc. [29]. Disturbance, cross-interference, caused by nontarget gases can occur because of the low selectivity of low-cost sensors [30]. These technical issues are difficult to solve as there are various solutions depending on the type and characteristics of the sensor. In this study, without addressing these technical issues, we used numerous low-cost homogeneous and heterogeneous sensors and utilized MOS sensors with poor cross-interference characteristics.

In addition to gas sensors, recognition algorithms are important elements in e-nose systems. The e-nose system employs various analytical techniques, including Principal Component Analysis (PCA), Discriminant Function Analysis (DFA), Support Vector Machine (SVM), K-Nearest Neighbor algorithm (k-NN), Random Forest (RF), Genetic Algorithm (GA), Multi-Layer Perceptron (MLP), and more [3–7, 12–17, 24–26]. The advantages of PCA include facilitating good visualization through dimensionality reduction, noise reduction, and enabling clustering of uncorrelated components. However, a disadvantage of PCA is its difficulty in interpreting the meaning of the principal component itself. Additionally, it cannot process nonlinear data and is sensitive to outliers [31, 32]. DFA offers high classification accuracy by effectively distinguishing classes and allows data reduction for improved visualization. However, a disadvantage of DFA is its potential poor performance due to vulnerability to nonlinear data and non-normal distributions within data classes [31, 32]. MLP has excellent non-linear modeling ability, can learn complex patterns through a multi-layer structure, and exhibits fast classification performance, especially with large amounts of data. Nonetheless, MLP has a long model training time, requires hyperparameter tuning, and may experience overfitting due to insufficient training data [31–33]. Considering the characteristics of these algorithms, both PCA and DFA were found suitable for the initial data review. Both algorithms allow for data visualization through dimensionality reduction and offer fast analysis performance. PCA can even cluster unclear samples, while DFA demonstrates excellent class classification performance between samples. Furthermore, it was determined that the MLP algorithm is the most suitable choice for classifying large amounts of training data and complex data patterns.

Various algorithms have been used to analyze odor patterns in e-nose systems. In the food field, Brudzewski et al. [3] classified the odor patterns of four different heat treatments of milk with 100% accuracy using the SVM algorithm. Okur et al. [4] used PCA, DFA, and the k-NN to distinguish six types of mint; the discrimination rates obtained were 97.2% (PCA), 100% (DFA), and 99.9% (k-NN). Ren et al. [5] used a Convolutional Neural Network to classify the freshness of 20 food products, which afforded an accuracy of 97.3%. Li et al. [6] used RF, MLP, SVM, and DFA algorithms to identify the flavors of Chinese liquor; the classification results were 100% (RF), 93.3% (MLP), 96.1% (SVM with sigmoid), 89.5% (SVM with linear), and 87.6% (DFA).

In the field of medical diagnostics, Pavlou et al. [12] classified urinary tract infections classes with 100% accuracy using a GA-NN algorithm. The GA-NN is a GA and MLP combined deep learning model to identify VOC patterns caused by bacterial contaminants in urine samples. Natale et al. [13] used a partial least-squares algorithm and classified lung cancer patients with 100% accuracy. Chen et al. [14] applied an MLP algorithm to classify lung cancer and normal patients, where the lung cancer patients were classified with 80% accuracy. Dutta et al. [15] used MLP, Probabilistic Neural Network(PNN) and SVM algorithms to classify six eye infection-causing bacteria in varying concentrations in saline solution, and the classification results were 75% (MLP), 94% (PNN), and 98% (SVM) accurate.

The olfactory sense of dogs is believed to be approximately one thousand times more sensitive than that of humans, based on the composition of biological olfactory receptors. When comparing the human olfactory system to that of a dog, dogs have significantly more olfactory receptors than humans, at least 30 times as many (150~300 million vs. 5 million). Additionally, dogs have approximately three times as many types of olfactory receptors (1,100 vs. 350). This implies that the number of olfactory receptors could be a more important parameter for classifying chemical compounds than the number of receptor types [34]. In this study, we propose an e-nose system consisting of multiple redundant sensors of the same type, similar to dog’s olfactory systems, to enchance the classification performance of chemicals without addressing the resolution and sensitivity issues of gas sensors encountered in existing e-nose research. The proposed e-nose system is based on numerous low-cost homogeneous and heterogeneous MOS-type sensors that exhibit cross-interference characteristics due to their low gas selectivity. The cross-interference characteristics among sensors of the same type are exploited by configuring numerous low-cost homogeneous and heterogeneous MOS-type gas sensors with similar selectivity. Thus, the multiple sensors yield similar yet slightly different signals due to variations in the dynamic characteristics of the low-cost sensors. The data obtained through multiple sensors are preprocessed into a form that can be learned via machine learning, and then analyzed using deep learning algorithms to evaluate the classification effectiveness of chemicals with respect to an increase in the number of homogeneous sensors.

**Fig 1:**
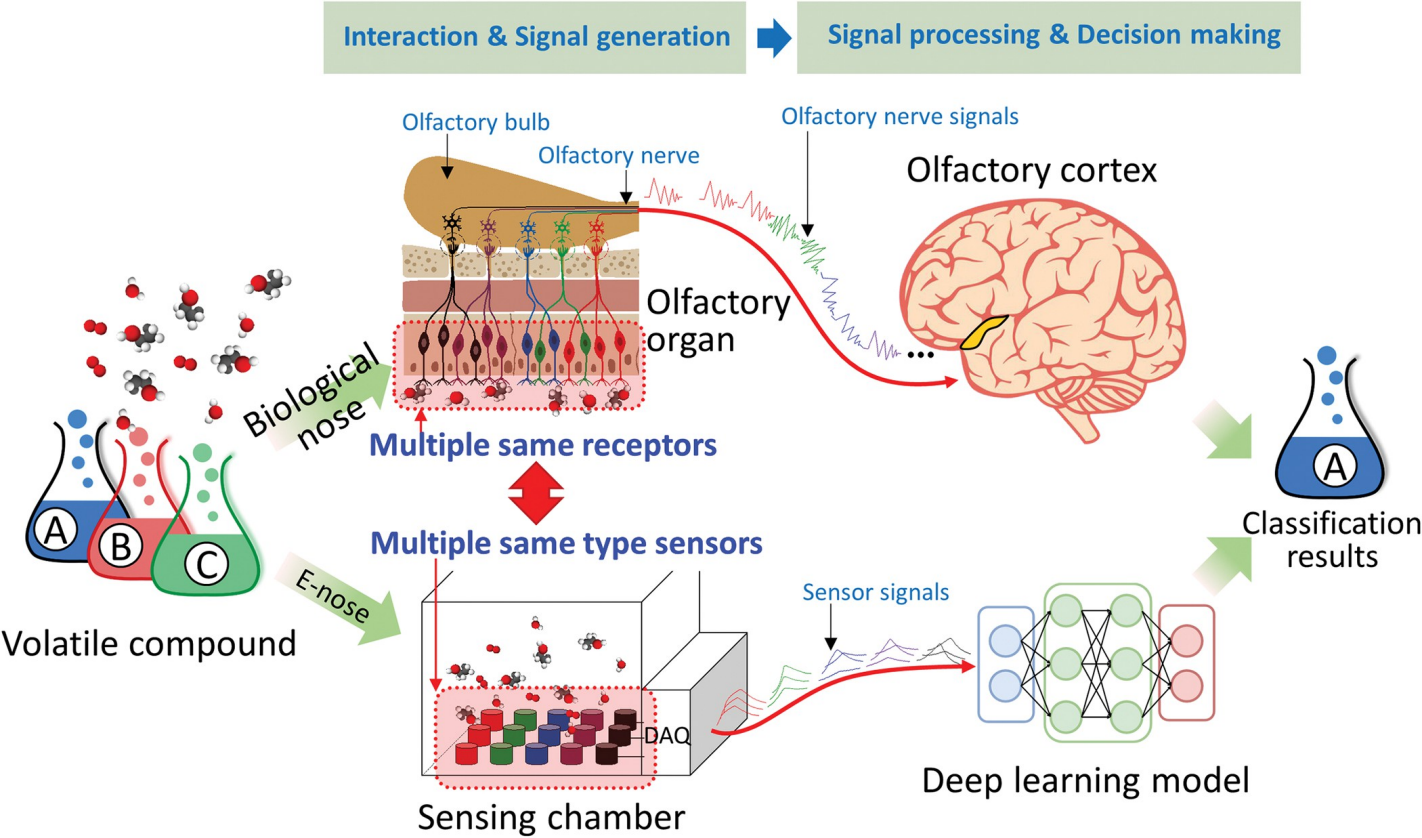
Comparison of human olfactory system and biomimetic electronic nose based on numerous homogeneous sensor arrays: Volatile compound classification.

Fig 1 shows a comparison of the classification of chemicals by the biological olfactory and e-nose systems. The upper section of Fig 1 shows the processing of odor particles in the biological olfactory system, which comprises many overlapping olfactory receptors with the same odor response characteristics. The olfactory signals produced by this organ are transmitted to the cerebrum via olfactory nerves. The transmitted olfactory signals are analyzed by the olfactory center of the brain, which analyses the odor pattern and classifies the odor. The lower section of Fig 1 shows the odor classification process of the proposed e-nose system that mimics the biological olfactory system. Numerous gas-sensor arrays with the same gas selectivity in an electronic system are used to mimic olfactory receptors in biological olfactory systems. The discretized gas sensor array signals are analyzed using a pretrained deep learning model that corresponds to the olfactory centers of the brain to classify odors by analyzing the odor signal patterns.

In this work, MLP algorithm is used. The MLP is an effective pattern analysis method for machine learning-based classifiers with nonlinear mapping capabilities and serves as an artificial neural network. This pattern analysis method, in addition to PCA, is the most typically used technique in the field of e-noses. An MLP comprises primarily three node layers, i.e., input, hidden, and output layers. The input layer receives the input signal to be processed. Necessary operations such as prediction and classification, are performed at the output layer. The arbitrary number of hidden layers placed between the input and output layers determines the performance of the MLP model [35]. Each node, except for the input node, is a neuron with a nonlinear activation function. The MLP is designed to approximate continuous functions. Multiple layers and nonlinear activation functions allow the MLP to distinguish between data that cannot be linearly separated [36]. In this study, rectified linear unit (ReLU) and scaled exponential linear unit (SeLU) activation functions are applied [37].

## Materials and methods

### Characterization of three VOC samples

**Fig 2:**
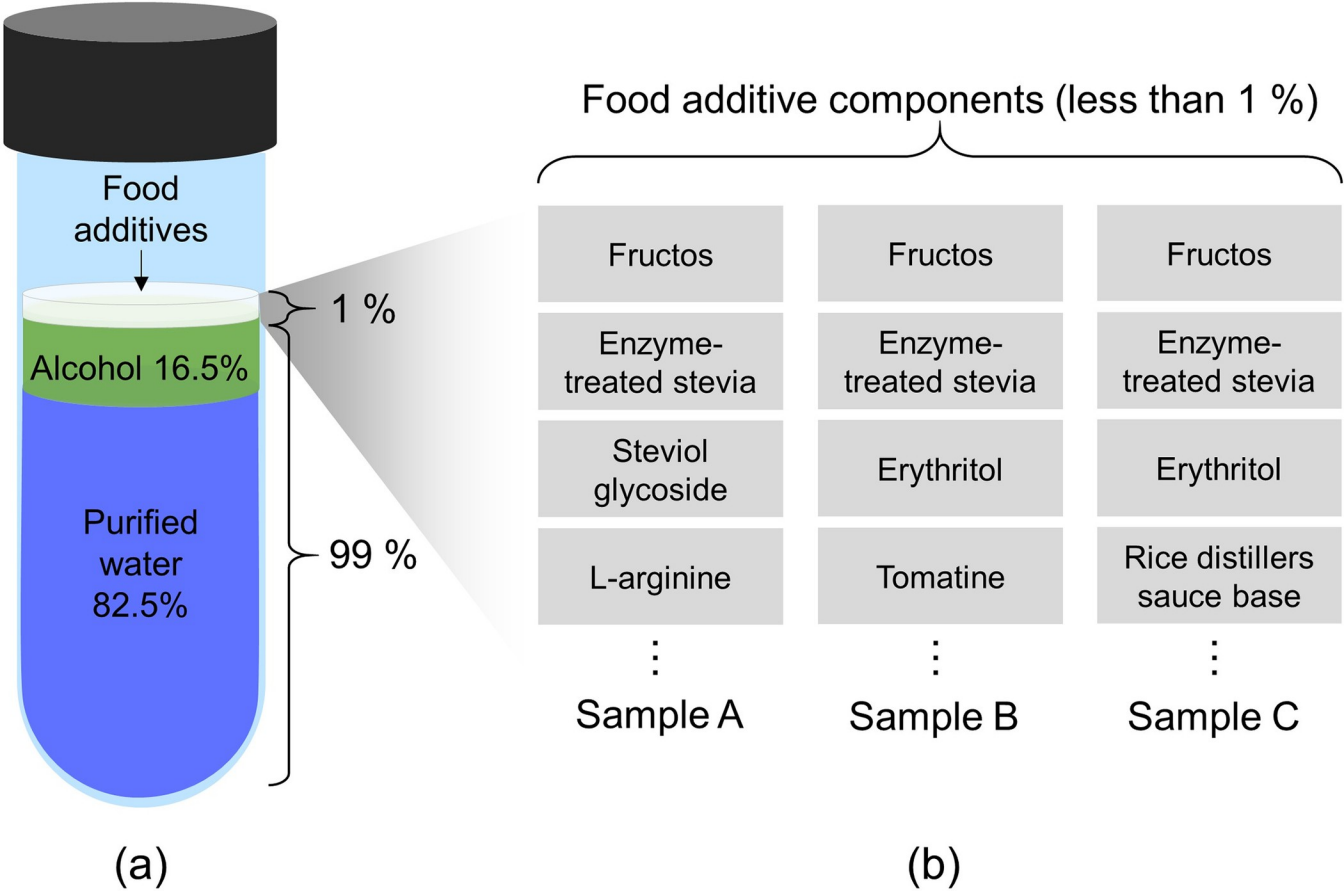
Components of samples that resulted in differences in VOC features. (a) Composition of sample solution; (b) composition of food additive for each sample.

**Fig 3:**
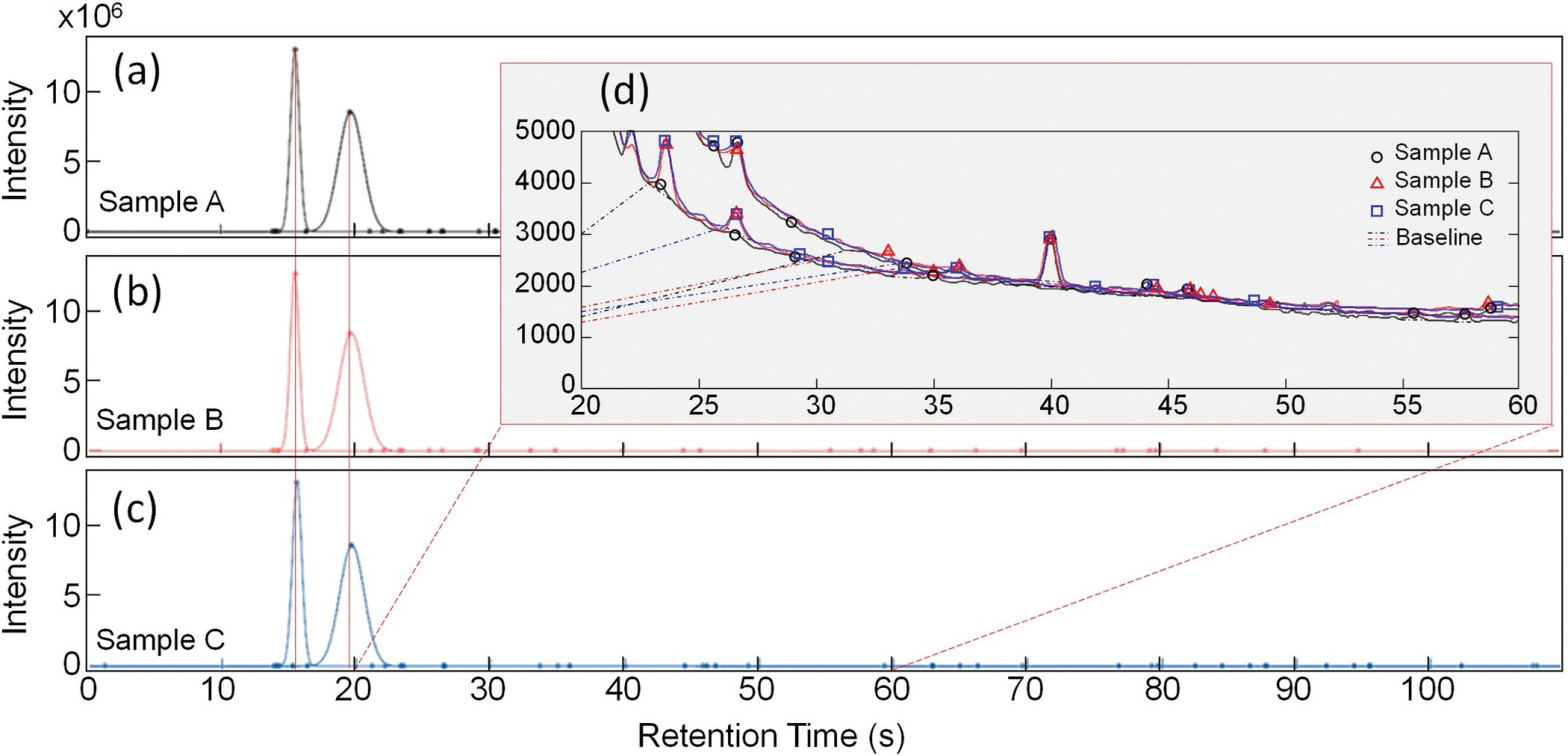
Result of volatile organic compound intensity obtained using FAST-GC-type commercial electronic nose system; Heracles Neo (AlphaMOS, France). (a)–(c) Intensities of samples A–C, respectively.

To obtain a dataset for the training and verification of chemical classifiers in a semiconductor sensor-based e-nose system, we prepared three commercially available diluted liquors (Chumchurum, Lotte Chilsung Beverage Co., Ltd., Republic of Korea; Chamisul-fresh, Hitejinro Co., Ltd., Republic of Korea; Jinro-is-back, Hitejinro Co., Ltd., Republic of Korea). Fig 2 shows the compositions of the three VOC samples that generated organic compounds in the experiment. As shown in Fig 2(A), 99% of the sample is composed of purified water and alcohol. Meanwhile, Fig 2(B) shows that approximately 1% of less of food additives contributed to the difference in organic compound generation among the VOC samples. Fig 3 shows the results of quantitative evaluation obtained using a commercial electronic nose system (Heracles Neo, AlphaMOS, France) with dual columns based on fast gas chromatography (FAST-GC) to compare the quantitative differences in organic compounds among the three VOC samples. To analyze the VOCs of the samples used, 5g of each sample was injected into a headspace vial (22.5 mm × 75 mm, PTFE/silicon septum, aluminum cap), and agitated at 40°C for 20 minutes at the speed of 500 rpm. Using an automated sampler, 1,000 μL of VOCs were obtained from the headspace. Subsequently, the samples were injected into a commercial chemical analysis system equipped with dual chromatographic columns of MXT-5 and MXT-1701, and subjected to analysis using a flame ionization detector. The analysis was conducted with the trap absorption temperature of 40°C, a trap desorption temperature of 250°C, and the acquisition time of 110 seconds.

**Fig 4:**
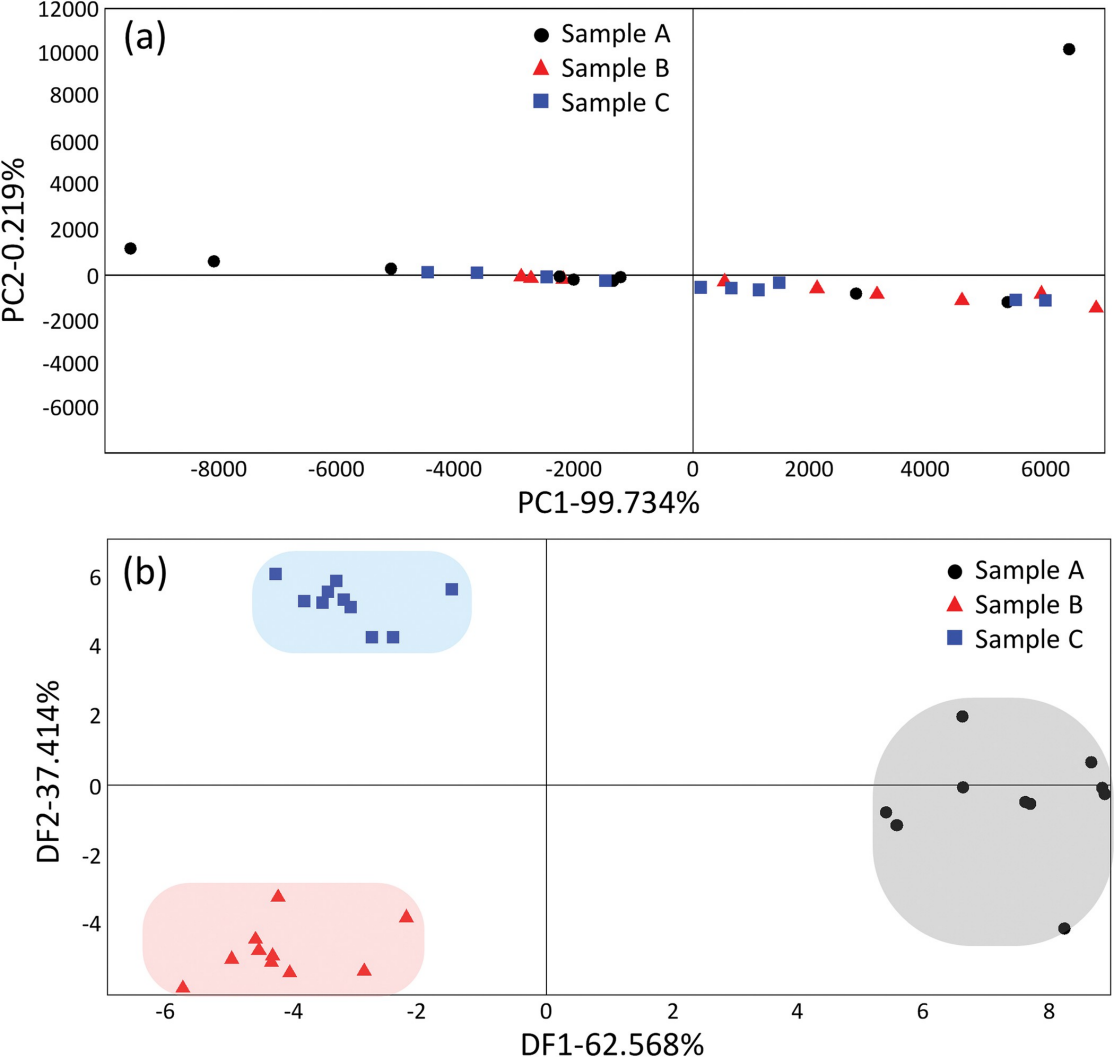
Results of classification among samples via statistical analysis using commercial electronic nose system, Heracles Neo (AlphaMOS, France); three types of VOC samples, 10 samples each. (a) Principal Component Analysis (PCA) result; (b) Discriminative Function Analysis (DFA) result.

In Fig 3(A)–3(C), the 15-s and 20-s bins along the X-axis indicate similar levels of intensity across the three samples. These intensity levels represent the peak intensity of the alcohol component, which is the dominant component in the VOC sample. Next, we compared the zoomed-in region between 20 and 60 s in Fig 3(D). The inset of each graph shows that the intensity of an extremely small fraction of the alcohol component varied for each sample; we used this difference in the organic compounds as the basis for classifying each sample. As shown in Fig 4, the three samples were measured 10 times each using the Heracles Neo e-nose system and analyzed using a statistical analysis program provided by the Heracles Neo e-nose system. Fig 4(A) presents the results of classification via PCA, which confirmed that the classification or clustering among the three samples was unsatisfactory. Fig 4(B) shows the results of classification via DFA, where a clear distinction was indicated among the three VOC samples. The results confirmed the quantitative differences in the organic compounds among the three VOC samples. This difference was confirmed by comparing the responses of a few columns other than that of the alcohol component. We confirmed that the classification performance varied significantly depending on the data classification model used. These results confirmed that the three prepared VOC samples differed from each other.

### Configuration of biologically inspired e-nose system

MOS-type gas sensors are the most widely used gas sensor technology in e-nose research; they offer the advantages of low cost, short response time, simple circuitry, and high persistence. However, it presents disadvantages such as low gas selectivity, high operating temperature, strong moisture effect, and nonlinearity at high temperatures [27]. The sensing materials such as SnO_2_, WO_3_, and ZnO, which are the main components of MOS-type gas sensors, have cross-interference characteristics that react to multiple gases simultaneously due to low gas selectivity [38]. This cross-interference is considered as the disadvantage in the chemical classification. However, cross-interference characteristics is positively used in the proposed system. Many low-cost homogeneous gas sensors were configured based on the numerous of biological olfactory receptors.

Table 1 lists the MQ gas sensor series (Hanwei Electronics Co., Ltd., China) applied to the e-nose system used in this study, and the sensors used in previous research literature on e-nose systems. Each gas sensor was a low-cost MOS-type gas sensor worth less than one dollar. Each low-cost MOS-type gas sensor exhibited high cross-interference characteristics. Notably, low-cost MOS-type gas sensors exhibit sensitivity deviations, even between sensors of the same target gas. These sensitivity deviations are caused by the inherent unevenness of the homogeneous products during production. These characteristics were used to redundantly configure the sensors to exploit the difference in the dynamic characteristics between each sensor as a feature of the odor pattern.

**Table 1:** List of gas sensors used in electronic nose system.

Model	Target gas (cross-interference)	Prices ($)	Manufacturer	Ref.
**MQ-2**	LPG, Propane, Hydrogen, Methane	0.6 ~ 1.9	Hanwei Electronics	This study
**MQ-3**	Alcohol, Benzene	0.7 ~ 1.9	Hanwei Electronics	This study
**MQ-4**	Methane, Alcohol, Hydrogen, LPG, LNG	0.6 ~ 1.9	Hanwei Electronics	This study
**MQ-7**	Carbon monoxide, Hydrogen, Methane, Alcohol, LPG	0.7 ~ 1.9	Hanwei Electronics	This study
**MQ-9**	Carbon monoxide, Methane, LPG	0.7 ~ 1.9	Hanwei Electronics	This study
**MQ-135**	Carbon dioxide, Carbon monoxide, Alcohol, Ammonia, Toluene, Aceton	0.7 ~ 1.9	Hanwei Electronics	This study
**TGS 822**	Acetone, Ethanol, Benzene, and Methane	5.2 ~ 12.2	Figaro Inc.	[3, 9, 16]
**TGS 826**	Ammonia, Ethanol, Hydrogen, and Isobutane	95.2 ~ 230.1	Figaro Inc.	[8, 16, 18]
**TGS 2600**	Methane, Isobutane, Ethanol, and Carbon Monoxide	5.2 ~ 7.6	Figaro Inc.	[9, 16, 19]
**TGS 2610**	Ethanol, Methane, Propane, and Isobutane	4.4 ~ 9.1	Figaro Inc.	[16, 19, 22]
**TGS 2620**	Carbon Monoxide, Ethanol, Isobutane, and Methane	10.4 ~ 33.0	Figaro Inc.	[9, 16, 19]
**DGS-CO 968–034**	Carbon Monoxide, Hydrogen, Isopropyl Alcohol	50.0 ~ 75.7	SPEC Sensors	[7, 11, 21]
**DGS-Ethanol 968–035**	Ethanol, Carbon Monoxide, Hydrogen Sulfide	50.0 ~ 75.7	SPEC Sensors	[7, 10, 11]
**DGS-SO2 968–038**	Sulfur Dioxide, Hydrogen Sulfide, Nitric Oxide	50.0 ~ 75.7	SPEC Sensors	[7, 20, 23]
**DGS-RESPIRR 968–041**	Nitrogen Dioxide, Hydrogen Sulfide, Ozone	50.0 ~ 75.7	SPEC Sensors	[7, 10, 11]

**Fig 5:**
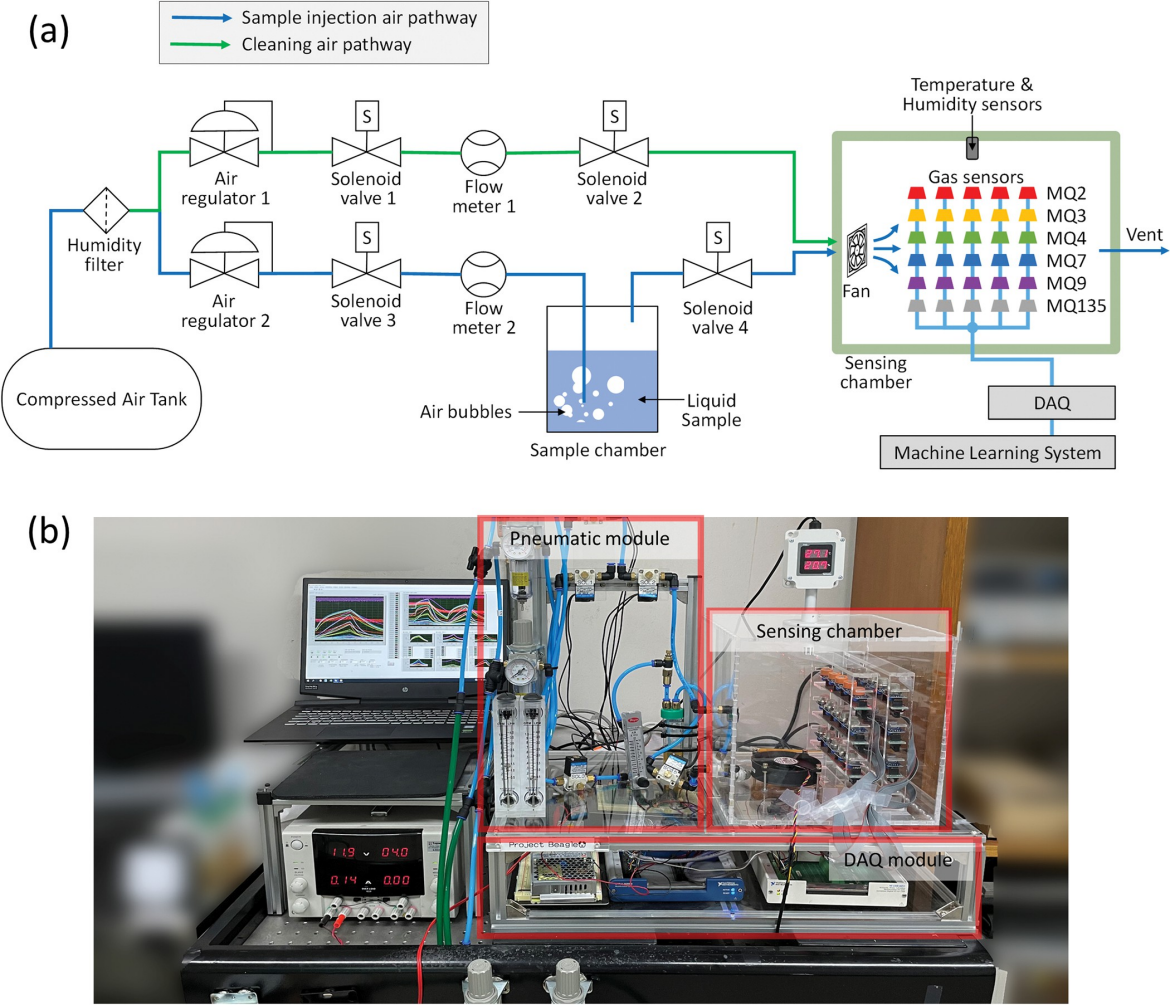
Schematic illustration of the proposed e-nose system. (a) Air pathway of bubbler based sample supply configuration; (b) photograph of the experimental setup.

The gas sensors used in this study were six types of heterogeneous sensors and five homogeneous sensors for each type with inherent different gas selectivity, and the sensor array was composed. Fig 5 shows a schematic diagram of the proposed e-nose measurement system. Fig 5(A) shows the pathway for cleaning the chamber containing the gas sensor array, and for feeding the sample into the chamber when measuring the dataset using the e-nose system. Fig 5(B) presents an example of an actual experimental setup. To acquire e-nose signals, a custom-built acrylic chamber with an internal volume of 250 mm × 250 mm × 250 mm was affixed with 30 sensors and thermohygrometer (THD-WD-1V, Autonics) to monitor the conditions inside the chamber. To ensure a stable voltage supply and to prevent voltage drops through the multi-sensors circuit configuration, a power supply (R-15-5, Meanwell) was configured individually for each sensor. A 90 mm fan (F129025SH, Everflow) was installed to facilitate air circulation in the chamber and was powered via a 12 V rated output. The automated sample supply system was controlled using two pneumatic regulators (SAW-2000, SKP pneumatic), one rotary flow meter (LZT M-15, Uxcell), one adjustable area flow meter (RMA-13-SSV, Dwyer instruments, Inc.), and four solenoid valves (KT30A-4G, KCC precision), which provided an air flow rate of 80.0 L/min for chamber cleaning and 0.6 L/min for sample supply. The temperature inside the chamber was 24°C ~ 35°C. This temperature distribution was determined by the airflow supplied to the sample cleaning section, which resulted in a temperature decrease within the chamber. The temperature of the chamber was maintained in the sample supply section. Pneumatic control and data acquisition were performed using two DAQ boards (NI-6251 and NI-6351, National Instrument) and a general-purpose laptop (17-CD0048TX, HP).

### Design of deep learning model

Data obtained from three different VOC samples using the proposed e-nose system were visually classified using PCA and DFA algorithms to verify the potential for classification, similar to the Heracles Neo e-nose system. Next, to evaluate the performance of the classifier based on the deep learning model, an MLP-based deep learning model was selected. The training dataset comprised six types of heterogeneous data and five homogeneous sensor data. Using the preprocessed data, a range of MLP model sizes and hyperparameters were selected to evaluate the effects of the cross-interference characteristics of the sensor array based on the number of configured homogeneous sensors. The experiments were conducted using a combination of three different neuron counts (1,000, 3,000, and 5,000 neurons), three different hidden layers (two, three, and four layers), and two different activation functions (SeLU and ReLU). We trained and validated 18 different combinations of machine learning models for each preprocessed data. For each model, three rounds of the training and validation are conducted, and then the classification accuracy is calculated as mean and standard deviation values. In the deep learning model, all parameters are fixed except the number of neurons and hidden layer, and activation function. For the fixed hyperparameters, we set the dropout to 0, kernel initializer to glorot, optimizer to Adam, learning rate to 0.00001, number of epochs to 200, and batch size to four. Figs 9 and 10 shows the visually classified results from PCA and DFA analysis, and the training and validation results from each MLP deep learning model and each preprocessing dataset, respectively. In training and validation of MLP model, 3,000 and 1,000 samples from each of the three types of VOC samples were used. The ratio of the training dataset to the testing dataset was 8:2. A stratified shuffle was performed to ensure no sample bias due to labeling during random sampling, followed by validation using the hold-out validation technique.

### Data measurement and preprocessing

**Fig 6:**
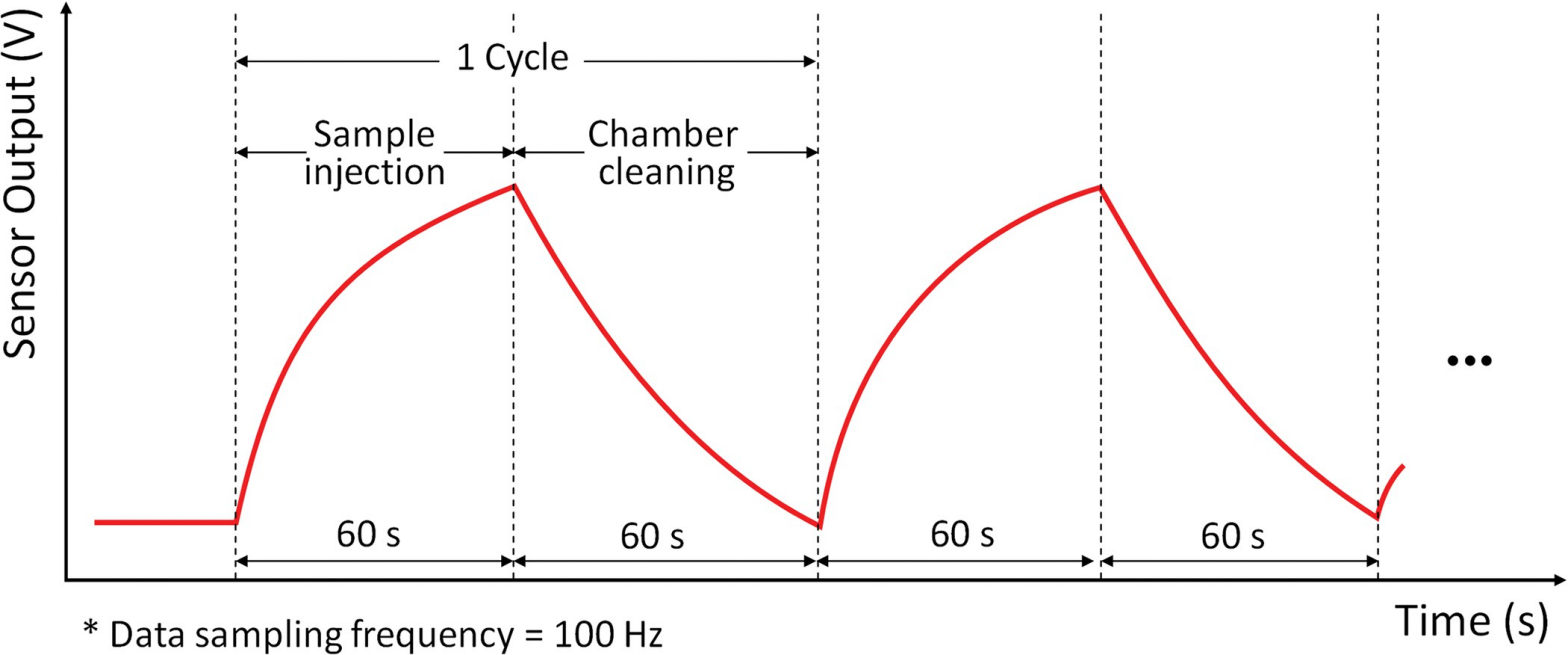
Data measurement process cycle of the e-nose system consisted of MOS-based gas sensors.

**Fig 7:**
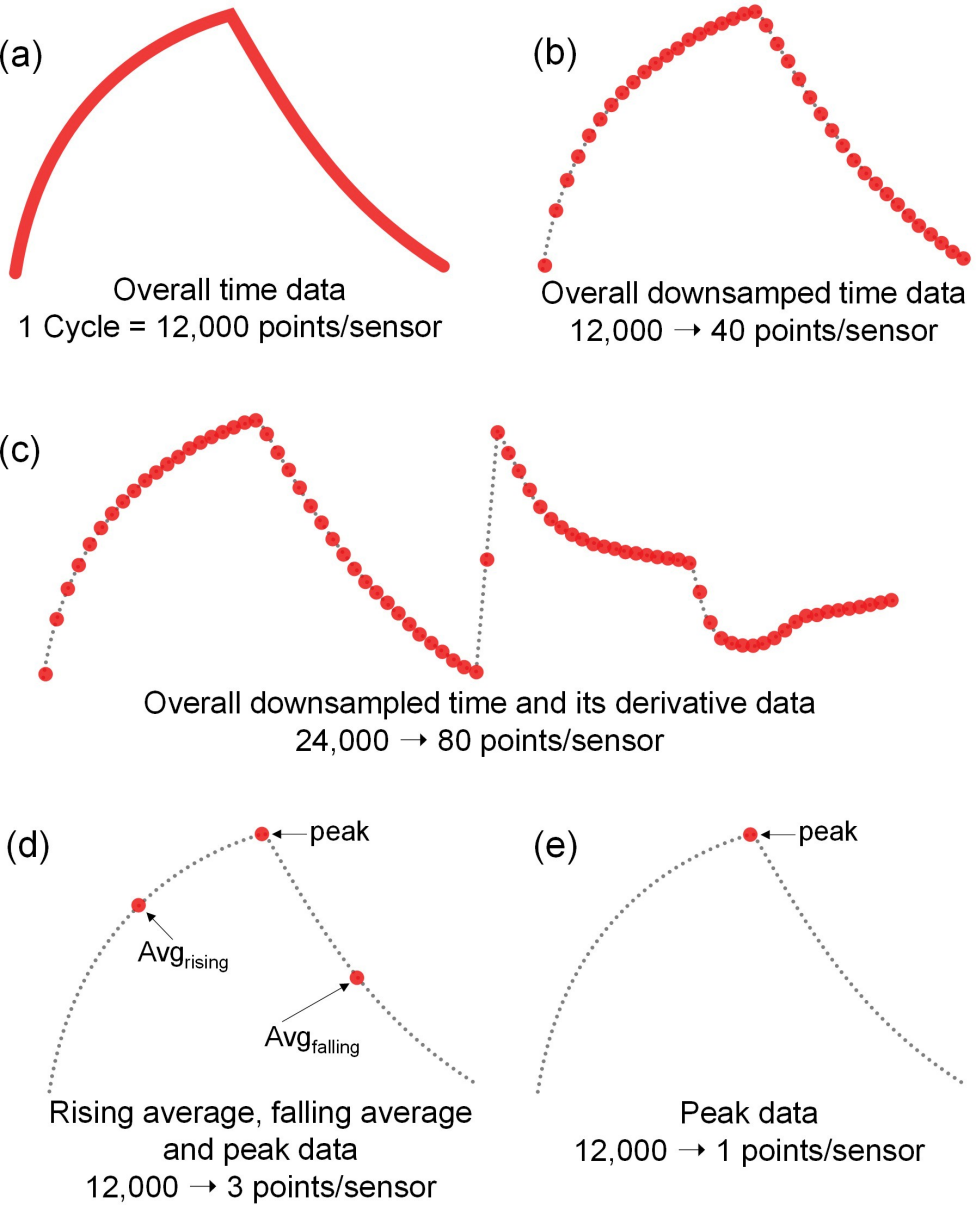
Data preprocessing methods for the deep learning model. (a) Overall original time data; (b) downsampled time data; (c) downsampled time and its derivative data; (d) rising average, falling average and peak data; (e) peak data.

**Fig 8:**
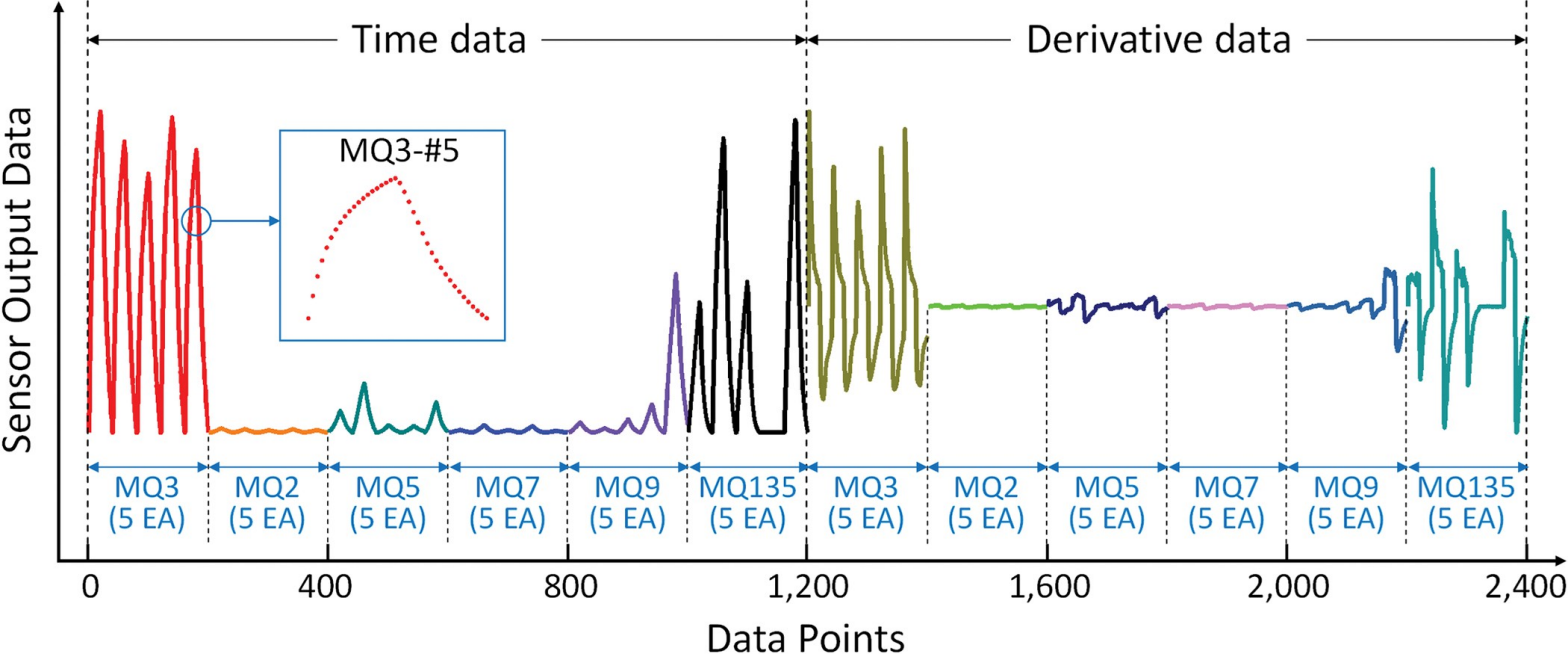
Dataset example of downsampled time and its derivative data obtained by 30 sensors (6 × 5 sensor array).

Fig 6 shows an example of the data measurement process for a gas sensor. As shown in Fig 5, clean air, which serves as the delivery gas, is directed into the sample chamber to vaporize the sample within the bubbler system. The sample vaporized by the bubbler was supplied for 60 s via the sample supply pathway, and the chamber was cleaned for 60 s via the cleaning pathway. Using the DAQ board, the data were measured at a sampling rate of 100 Hz for 120 s, which resulted in 12,000 data points per sample. In our preliminary experiment, we initially checked the sampling period of gas sensors for up to 5 minutes, and then we determined that 1 minute was enough to distinguish samples. To utilize machine learning, the data acquired in the form of time-domain response curves were preprocessed using several techniques. The following eight techniques [39] are typically used to preprocess time-series data: peak response, response in special time, time in special response, area, integral, derivative, difference, and second derivative. In this study, time-domain data were used as a basis for evaluating the performance of each pretreatment, and three types of feature extraction techniques were applied to construct the pretreatment data, including the peak response, response in special time, and derivative methods. Fig 7 shows five different data forms preprocessed from the raw time-domain data obtained via the process shown in Fig 6. Fig 7(A) and 7(B) shows the original time-domain data, and overall downsampled time-domain data (300:1 ratio), respectively. Fig 7(C) is combination data of overall downsampled time-domain data and the its derivative data. Fig 7(D) shows three data points including the average value of the sample supply section, the peak value of the sensor response data, and the average value of the chamber cleaning section. Fig 7(E) shows only the peak values of the sensor response data. Fig 8 shows an example of the data generated via the preprocessing of Fig 7(C), where the 300:1 downsampled data and derivative data of six types of heterogeneous sensors and five homogeneous sensors are organized into a dataset.

## Results and discussions

### Characterization of preprocessing data

**Fig 9:**
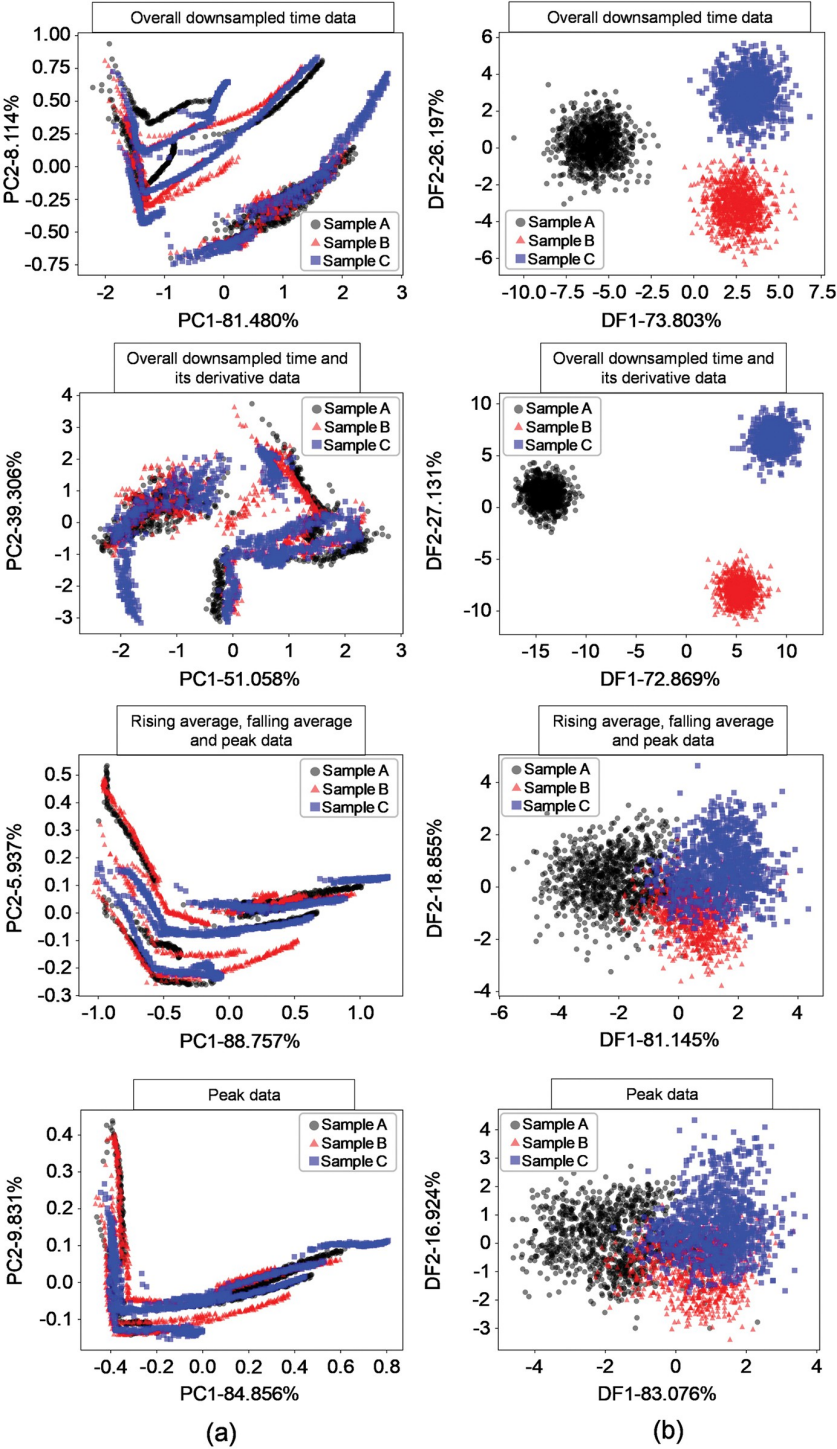
Statistical analysis results according to data preprocessing methods. (a) PCA results; (b) DFA results.

As shown in Fig 9, we examined the data using PCA and DFA methods to determine whether the preprocessed data can be classified accurately via machine learning. In Fig 9, each row shows the PCA and DFA results of four different preprocessing data forms in Fig 7. Fig 9(A) shows the result of PCA classification of each preprocessed data form, which indicates no clear distinction between the sample classes. On the other hand, Fig 9(B) shows the results of DFA classification of each preprocessed dataset. The results show that the sample groups were discriminated relatively well. Specifically, the overall downsampling time data and the overall downsampling time data + derivative preprocessing data were clearly clustered in the DFA results. These results show a similar trend to the preliminary results of the characterization of the three VOC samples investigated by Heracles Neo e-nose system in Fig 4. Therefore, we speculate that the samples can be distinguished when performing machine learning.

### Hyperparameters and data preprocessing effect

**Fig 10:**
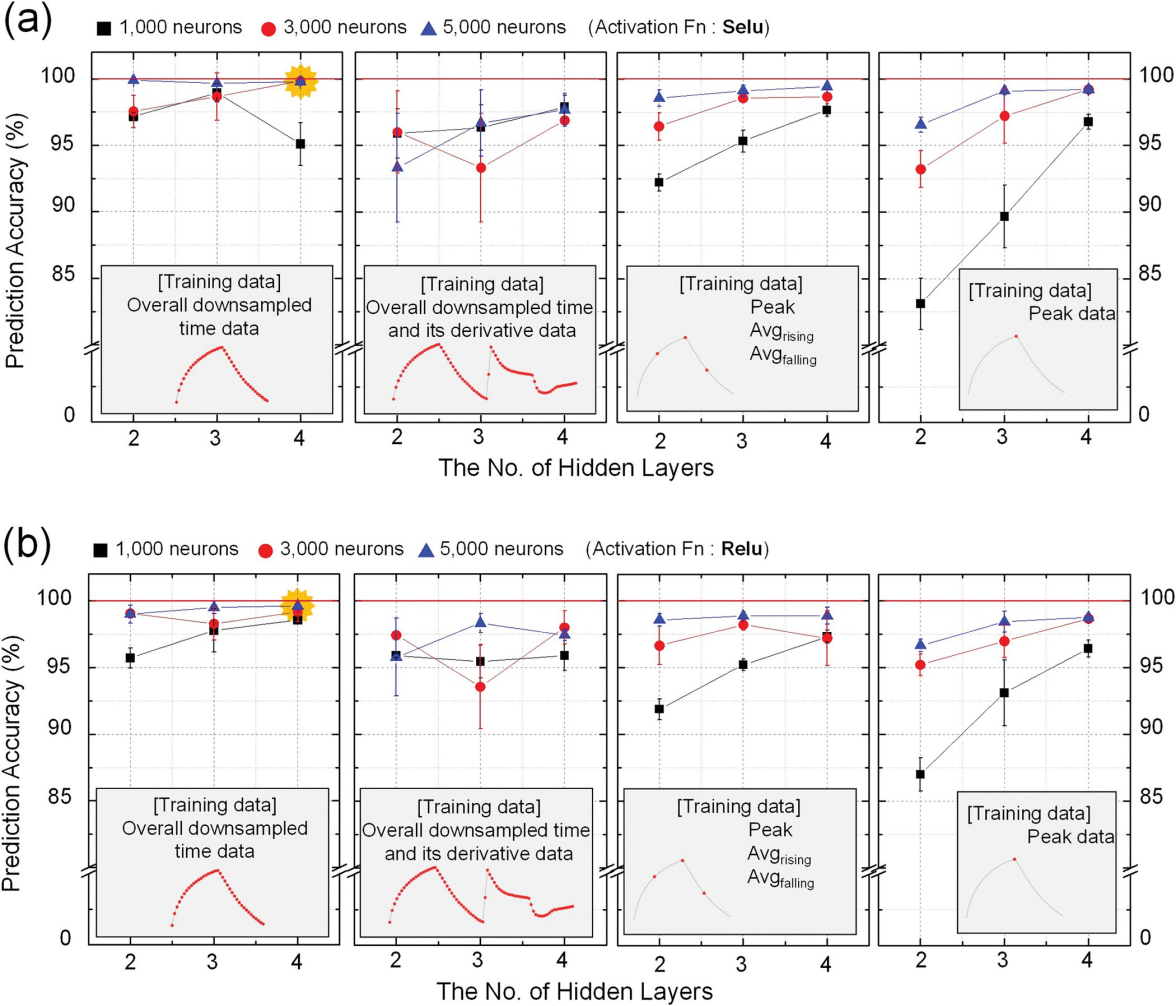
Result of classification accuracy according to data preprocessing methods and hyper-parameters in deep learning model (6 × 5 sensor array); (a) SeLU activation function; (b) ReLU activation function.

Fig 10 shows the prediction accuracy of MLP based deep learning model for different data preprocessing methods. Depending on the data preprocessing methods of (1) downsampled time data, (2) downsampled time and derivative data, (3) rising and falling average and peak data, and (4) peak data only, the prediction accuracy showed up to 99.61 ± 0.07% for 5,000 neurons, 4 hidden layers and Selu function, 97.67 ± 1. 25% for 5,000 neurons, 3 hidden layers and Relu function, 99.44 ± 0.16% for 5,000 neurons, 4 hidden layers and Selu function, and 99.22 ± 0.16% for 5,000 neurons, 4 hidden layers and Selu function, respectively. In the classification models of PCA and DFA, the combination of downsampled time and its derivative data presents best classification results as shown in Fig 9, while in the prediction model based on MLP, downsampled time data without derivative data shows higher prediction accuracy rather than the average and peak data. The experimental results confirmed that the SeLU activation function exhibited excellent performance and that the classification performance differed despite the different data dimensions (i.e., 1200, 2400, 90, and 30 points). In general, the classification performance improved as the number of hidden layers and neurons increased. Under both activation functions, the highest classification performance was observed when the average and peak data were combined. To evaluate the classification performance of the proposed e-nose system, we selected SeLU as the activation function, four hidden layers, and 5,000 neurons.

### Effect of the number of sensors

**Fig 11:**
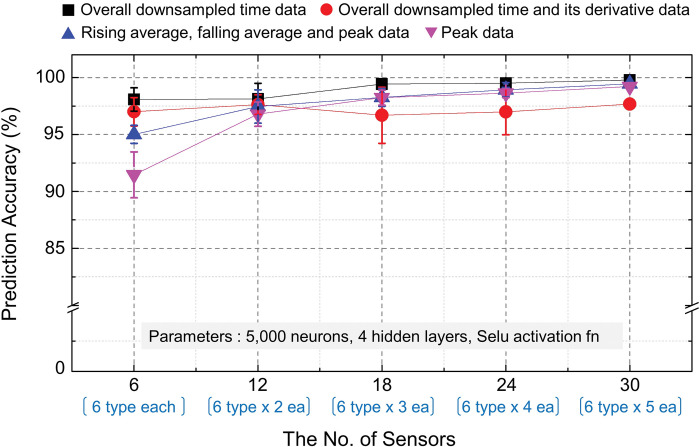
Classification accuracy of the proposed electronic nose system with respect to the number of homogeneous sensors. (MLP: four hidden layers, 5,000 neurons, SeLU activation function).

The classification performance of the proposed e-nose system was evaluated with respect to the number of homogenous sensors. Four different preprocessed data forms obtained from 30 sensors (six types of heterogeneous sensors and five homogeneous sensors for each type) were reconstructed. The number of homogeneous sensors was reduced one by one. Thus, five different cases were prepared, such as six different types of heterogeneous sensors each (6 × 1 sensor array), six types of sensors with two sensors of the same type (6 × 2 sensor array), and six types of sensors with five sensors of the same type (6 × 5 sensor array). The training and validation conditions of the deep learning model were the same as described before. Fig 11 compares the classification accuracy of three VOC samples in the proposed e-nose system, considering both data preprocessing methods and the number of homogeneous sensors. Generally, the classification accuracy increased as the number of homogeneous sensors increased.

For overall downsampled time data, when increasing the number of homogeneous sensors from one to five, the classification accuracy of the e-nose systems was increased from 98.08% to 99.78%. In the cased of the combination of overall downsampled time data and derivative data, the classification accuracy of the e-nose systems was increased from 97.00% to 97.67%. For the rising average, falling average and peak data, the classification accuracy of the e-nose systems was increased from 95.01% to 99.44%. When only peak data was used, the classification accuracy of the e-nose systems was increased from 91.46% to 99.22% as the homogeneous sensors were increased from one to five.

## Summary and conclusion

In this study, an e-nose system based on numerous low-cost homogeneous and heterogeneous sensors configured based on the arrangement of biological olfactory receptors was proposed. The proposed method only increases the feature count of sample data to enhance recognition accuracy, without addressing the primary issues affecting sensor performance. The system positively used the cross-interference characteristics of MOS type gas sensor due to their low gas selectivity. Furthermore, the effect of preprocessed data forms in deep-learning model on enhancing the classification performance of the e-nose system was investigated. From DFA classification results of the three VOCs samples based on the preprocessed data, overall downsampled time and its derivative data shows a clear distinction. Meanwhile, the overall downsampled time data was more effectively worked in deep learning model. For the overall downsampled time data, the classification accuracy was increased up to 99.78%. The highest overall performance was achieved using the SeLU activation function, four hidden layers, and 5,000 neurons. We confirmed that the classification performance improved as the number of hidden layers and neurons increased. Based on these results, the classification accuracy of the MLP model was evaluated for the different number of homogeneous sensors. Six types of heterogeneous sensor were fixed, and then the number of homogeneous sensors was changed from one to five. The results showed that using numerous homogeneous sensor array configurations improved classification performance. For overall downsampled time data, the best performance achieved by 6 × 1 sensor array (240 data points) was 98.0%, and by 6 × 5 (1,200 data points) sensor array was 99.78%. For overall downsampled time and its derivative data, the best performance achieved by 6 × 1 sensor array (480 data points) was 97.00%, and by 6 × 5 (2,400 data points) sensor array was 99.67%. For rising average, falling average and peak data, the best performance achieved by 6 × 1 sensor array (18 data points) was 95.01%, and by 6 × 5 (90 data points) sensor array was 99.44%. In the case of peak data (only 1 data point per sensor), the best performance achieved by 6 × 1 sensor array (6 data points) was 91.46%, and by 6 × 5 (30 data points) sensor array was 99.22%. This result confirmed that redundant sensor arrays with the same type of sensor can improve the classification performance with extremely few data points. From the experimental results, we verified that the electronic nose systems configured by only numerous low-cost sensors without expensive high precision sensors could successfully classify similar VOCs samples like commercial electronic nose systems of Heracles Neo.
